# From data to wisdom: harnessing the power of multimodal approach for personalized atherosclerotic cardiovascular risk assessment

**DOI:** 10.1093/ehjdh/ztad068

**Published:** 2023-11-18

**Authors:** Sadeer Al-Kindi, Khurram Nasir

**Affiliations:** Center for Cardiovascular Computational and Precision Health (C3PH), DeBakey Heart and Vascular Center, Houston Methodist, 6550 Fannin Street, Suite 1801, Houston, TX 77030, USA; Center for Cardiovascular Computational and Precision Health (C3PH), DeBakey Heart and Vascular Center, Houston Methodist, 6550 Fannin Street, Suite 1801, Houston, TX 77030, USA

**This editorial refers to ‘Improving cardiovascular risk prediction through machine learning modelling of irregular repeated electronic health records’, by C. Li *et al*., https://doi.org/10.1093/ehjdh/ztad058.**

The influx of data in medical practice has soared, spanning a vast range from electronic health records to diagnostic imaging to genomics. Despite plethora of data at our fingertips, it is astonishing how limited our scope of application remains. Data represent the bottom layer of understanding in the information pyramid, and information, knowledge, and finally intelligence and wisdom build upon data in ascending levels of comprehension and insight. Deliberate analysis and interpretation are thus required to convert raw facts into contextualized knowledge, wisdom, and actionable steps, which can predict future outcomes, guide evidence-based care, and improve population health.^1^

Clinical practice has thus far seen only limited utilization of these rich data sources. The capacity for multidimensional thinking is bounded by inherent limitations of human cognition. Consequently, even our most sophisticated risk scores and predictive models seem rudimentary. Outside of medicine, many fields have seen rapid advances thanks to computational analytics. From online recommendations to financial modelling, industries have been revolutionized by repeatedly mining large datasets for patterns and insights that optimize practices. However, medicine has lagged behind in embracing these techniques, partly due to the challenges posed by medical data complexity. This variability and disorder make applying traditional statistical methods difficult. There is, however, tremendous potential for computational approaches to similarly transform medical practices.

A glaring manifestation of these current limitations is evident in the prediction of atherosclerotic cardiovascular disease (ASCVD). Almost exclusively, these risk models focus on a narrow set of data points, neglecting the wealth of insights lying at various intersections of patient data.^2^ Often, these models are static, reducing their efficacy as they fail to capture the dynamic progression of a patient’s health. More sophisticated techniques are needed to uncover multidimensional relationships, interdependencies, and temporal dynamics. As we continually add to our therapeutic arsenal, ensuring that interventions are as precise as possible, treating the right patient at the right time becomes paramount. The urgency for enhanced predictive analysis for ASCVD risk is not just a matter of technological progress; it is a necessity driven by a confluence of patient needs, economic imperatives, and the overarching goal of improving health outcomes within a resource constraint environment.^3^

In this issue of EHJ Digital Health, Li *et al*.’s^4^ study of more than 200 000 adults (40–79 years) without ASCVD in China illuminate the way forward. The authors followed individuals longitudinally and analysed demographic data, and 25 repeated measures of anthropometric measures and vital signs (e.g. weight, blood pressure), blood markers (lipids, glucose, renal function), medications to predict ASCVD risk (defined as myocardial infarction, stroke, and death due to stroke or coronary artery disease). Summary statistics (such number of measurements, variance, and trajectories) were utilized as model inputs. The investigators used two supervised models, eXtreme Gradient boosting and least absolute shrinkage and selection operator regression, to predict the 5-year incidence of ASCVD events. Overall, the machine learning models achieved good discrimination of ASCVD events (c-statistic of 0.79) and improved discrimination, calibration, reclassification, and clinical utility (measured by decision-curve analysis) as compared with guideline-recommended clinical model based on regression (China-PAR). While the absolute differences in these metrics, as compared with clinical models, are small, they can have large impact when applied to large populations.

**Figure 1:**
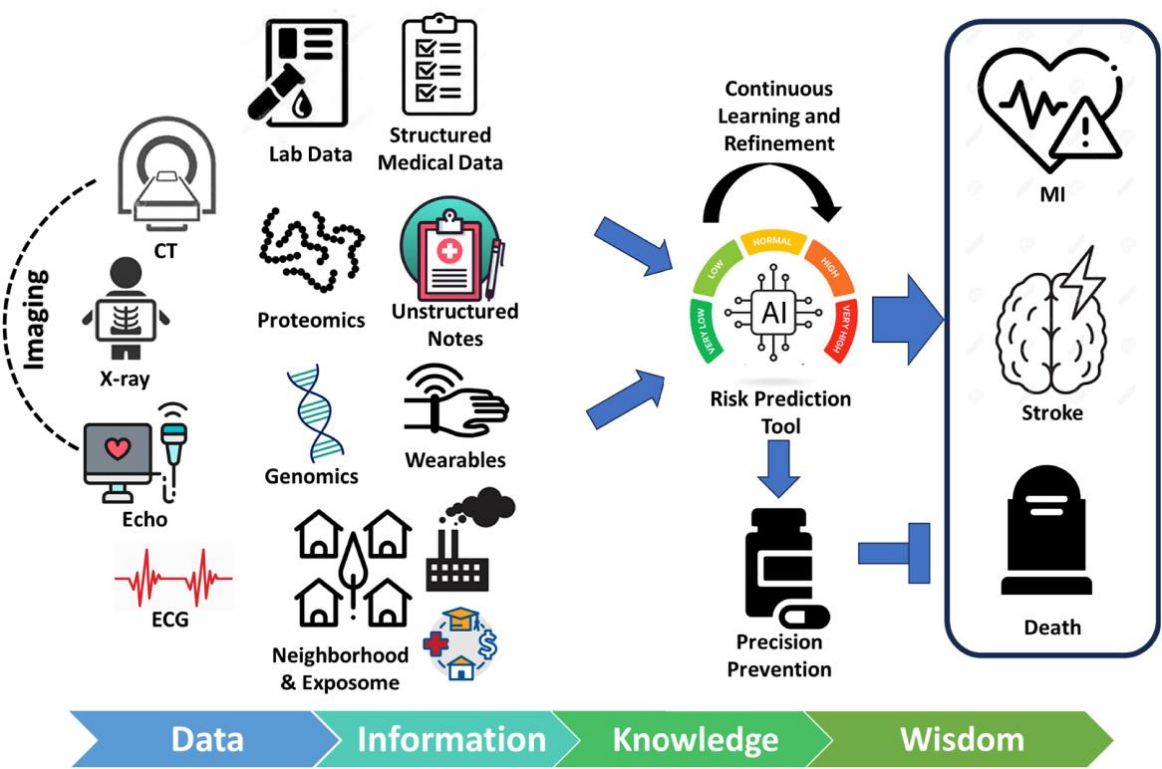
Multimodal computational platforms for precision prediction of cardiovascular risk across the knowledge continuum.

As we applaud Li *et al*.’s efforts, these efforts reflect just the beginning. Looking forward, further refinement of risk prediction will require incorporating diverse multimodal data sources beyond the limited International Classification of Diseases codes, vitals, procedures, labs, and medications. Such a narrow viewpoint hinders our ability to truly understand and predict complex diseases like ASCVD. Structured clinical data, though immensely valuable, is just the tip of the iceberg. Beneath this is a depth of unstructured data in the form of clinical notes, reports, and imaging findings represent an untapped opportunity when combined with natural language processing.^5^ Advanced imaging biomarkers and those derived from biosignals, such as ECG-based risk scores,^6^ can provide additional dimensions. Linking social determinants of health^7^ and environmental factors^8–10^ with clinical data could also offer unique risk insights (*Figure 1*). Using proxies like a patient’s home residential address, for example, can yield invaluable data on factors such as social vulnerabilities, area deprivation, air pollution, and other environmental exposures. These elements, often sidelined in traditional analyses, play a profound role in influencing ASCVD risk predictions. Future advancements in risk prediction clear hinge on assimilating diverse data sources. Additionally, longitudinal tracking of risk trajectories over time using timeseries data could unlock new temporal dynamics.^11^ With exponential growth in computational power, advanced analytics applied to interconnected, multimodal patient data at scale will enable a new paradigm of preventive medicine with unprecedented personalization and precision.

But ‘with great power comes greater responsibility’. While confluence of medical data and computational prowess offers untapped potential in reshaping the landscape of cardiovascular disease risk prediction, it is imperative to also exercise caution and rigour. New techniques might unveil potential risk factors previously uncharted and unprecedented depth in data analysis, they do not negate the need for human expertise. Although machine learning is adept at identifying subtle correlations in datasets, we must remember that correlation is not causation. We must strive to understand the foundational reasons for these correlations to ensure their relevance and appropriateness.

In conclusion, the pioneering work of researchers like Li *et al*. has shown us the direction that with determination, innovation, and the right tools, we can navigate these hurdles. However, we would like to also remind that this transformation does not need to be just a technical one; it is imperative that it is deeply human. The future of these efforts has to be about harnessing the power of data and technology to impact lives, alleviate suffering, promoting health, and enhance resource utilization. Every byte of data, algorithm, and predictive model should serve a singular purpose: to enhance the human experience of both the caregiver and the receiver. Adhering to this fundamental principle, while harnessing the full potential of our data resources and integrating them with cutting-edge computational methods, we can set the stage for a new era of medicine—one that is truly personalized, proactive, and precise, but also centred on human values and connections.
